# Spin-Hall-Effect-Assisted Electroresistance in Antiferromagnets via 10^5^ A/cm^2^ dc Current

**DOI:** 10.1038/srep31966

**Published:** 2016-08-22

**Authors:** Jiahao Han, Yuyan Wang, Feng Pan, Cheng Song

**Affiliations:** 1 Key Laboratory of Advanced Materials (MOE), School of Materials Science and Engineering, Tsinghua University, Beijing 100084, China

## Abstract

Antiferromagnet (AFM) spintronics with reduced electrical current is greatly expected to process information with high integration and low power consumption. In Pt/FeMn and Ta/FeMn hybrids, we observe significant resistance variation (up to 7% of the total resistance) manipulated by 10^5^ A/cm^2^ dc current. We have excluded the contribution of isotropic structural effects, and confirmed the critical role of the spin Hall injection from Pt (or Ta) to FeMn. This electrical current-manipulated resistance (*i.e*. electroresistance) is proposed to be attributed to the spin-Hall-effect-induced spin-orbit torque in FeMn. Similar results have also been detected in plain IrMn films, where the charge current generates spin current via the spin Hall effect with the existence of Ir atoms. All the measurements are free from external magnetic fields and ferromagnets. Our findings present an interesting step towards high-efficiency spintronic devices.

The burning requirement for high-efficiency information processing advances spintronic devices with low power consumption and high-density integration^1^. Although magnetic random access memory (MRAM) based on ferromagnets (FMs) have been widely used in data recording^1^,^2^,^3^, the employment of external Ørsted fields in MRAM consumes remarkable electrical energy^4^. Besides, the existence of FM stray fields hinders the integration density from increasing^5^. Owing to the compensated magnetic moments, antiferromagnets (AFMs) show significant advantages over its FM counterpart: free from stray fields, rigid to magnetic field perturbations, and possibly having stronger spin torques for better control^6^,^7^,^8^. In this scenario, AFMs are greatly expected to perform low-power and high-integration spintronics without external fields and FMs.

To build an available AFM-based device, it is essential to explore observable effects manipulated by AFM order parameters. The discovery of tunneling anisotropic magnetoresistance in AFMs possibly fulfills the demands, whereas the AFM moments are dragged passively by a neighboring FM layer via external fields^9^,^10^,^11^. Moreover, current-influenced AFM interactions have been detected in AFM/FM hybrids^12^,^13^, but high-frequency ac current and FMs are inevitable for signal detection. Recently, Wadley *et al*. discovered the electrical control of magnetoresistance in antiferromagnetic CuMnAs, where the rotation of AFM moments was induced by 10^6^ to 10^7^ A/cm^2^ pulsed current^14^. As it is generally accepted that the threshold current of semiconductors is of 10^5^ A/cm^2^ magnitude^1^,^2^, direct scheme of manipulating spin configuration in AFMs via low-density dc current is particularly urgent.

Fortunately, an emerging term of spin torques, which originates from spin-orbit coupling and is thus named by spin-orbit torque (SOT), makes the task achievable. The SOTs have already been engineered to invert FM moments, leading to current manipulation of magnetization in FMs^15^,^16^,^17^,^18^,^19^,^20^,^21^. However, due to the rigid arrangement of AFM moments, the torque generated by moderate dc current in AFMs seems too weak to induce detectable rotation of the order parameters. Assisted by the spin injection via the spin Hall effect (SHE)^15^,^17^,^18^, the SOT would exert significant influence on non-equilibrium magneto-transport in AFMs. In this way, we observe remarkable electroresistance (up to 7% of the total resistance, defined as the resistance change when applying a large current compared to the resistance with a certain small current) in Pt/FeMn, Ta/FeMn, and IrMn ultrathin films via 10^5^ A/cm^2^ dc current. Owing to the extensive application of these metallic AFMs and the low-density dc current, our work may lead to low-power and high-integration information processing, and build up a bridge between AFM spintronics and semiconductor electronics.

## Results

**Figure 1:**
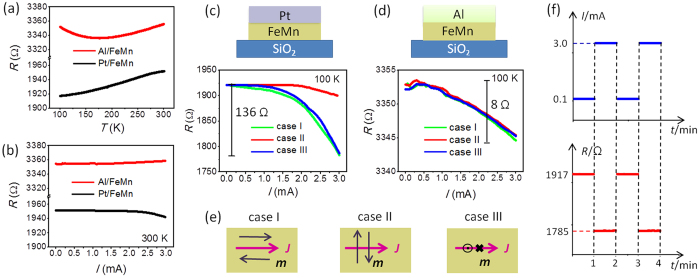
Electroresistance in Pt(2)/FeMn(7) and Al(4)/FeMn(7). (**a**) Resistance–temperature (*R*–*T*) curves of Pt/FeMn and Al/FeMn measured with an applied current of 100 μA. (**b**) Resistance–current (*R*–*I*) curves of Pt/FeMn and Al/FeMn measured at 300 K. (**c**,**d**) *R*–*I* curves of Pt/FeMn and Al/FeMn after field cooling from 300 to 100 K with a magnetic field of 1 T. The data were measured at 100 K after repealing the cooling field. (**e**) Definition of the field cooling modes (namely case I, II and III). (**f**) Response and stability of the electroresistance in Pt/FeMn.

We first study the current-induced electroresistance in Pt(2)/FeMn(7) bilayers (unit of thickness: nanometer). The films have been fabricated into Hall-bars with width of 50 μm, and the distance between the two side electrodes which were used to measure the longitudinal resistance (*R*) is 400 μm. The resistance of the sample presents a positive temperature–resistance (*R*–*T*) behavior from 100 to 300 K (Fig. 1a), and remains nearly constant with the increasing current at 300 K (Fig. 1b). The sample was cooled from 300 to 100 K with a magnetic field of 1 T along the current direction. Unlike vertical alignment of AFM moments forced by a strong magnetic field (*i.e*., the spin-flop transition), a cooling field lower than the threshold value, which is determined by the material itself, leads to collinear alignment^22^,^23^. In most studies on FM/AFM hybrids, the field-cooling process creates an AFM easy-axis collinear with the cooling field with the assistance of interfacial exchange coupling^9^,^10^,^11^. The exchange bias in Figure S1 suggests parallel orientation between the cooling field and the AFM moments in FeMn. Recently with growing attention to AFM spintronics, it has been demonstrated that the cooling field lower than the threshold value also creates collinear alignments of AFM moments in plain AFM layers (*e.g*., Cr_2_O_3_, IrMn)^24^,^25^. The threshold field is quite large for most AFMs, for example, 6 T for Cr_2_O_3_^26^. We also calculate the threshold field^23^ of FeMn given as 2(*H*_an_ · *H*_mole_)^1/2^. *H*_an_ is the anisotropic field and *H*_mole_ is the molecular field. *H*_an_ of FeMn is 5 mT^27^. *H*_mole_ is estimated by the value of Fe as 10^3^ T. The threshold field of spin-flop transition would be 2(5 mT · 10^3^ T)^1/2^ = 4.5 T, far above our cooling field of 1 T. Hence, the cooling field can create an AFM order arranged along *x*-axis dominantly, as case I in Fig. 1e.

The resistance–current (*R*–*I*) measurements were carried out at this temperature after removing the field. The most striking result here is the negative electroresistance (resistance from 1921 to 1785 Ω, ~7% variation of the total resistance) when the current increases from 0.005 to 3 mA, the limit of our current source (case I in Fig. 1c), corresponding to relatively low current density of 3 mA/[50 μm × (2 + 7)nm] ~ 10^5^ A/cm^2^. Interestingly, this value satisfies the demanding of semiconductor industry^1^,^2^. The *R*–*I* curve shows almost no difference if the field of 1 T was maintained during the measurements, reflecting the rigidity of AFMs to magnetic field perturbations^14^. And the resistance presents very similar behaviors with negative current, which is shown in Supplementary Figure S3. Results with similar trends are obtained in another Pt(2)/FeMn(7) sample fabricated and measured in the same conditions, which suggests the reproducibility of this effect (Supplementary Figure S3). We further examine the response and stability of the current-induced electroresistance. A constant current of 0.1 and 3.0 mA is applied to Pt/FeMn alternately every minute. Correspondingly, the resistance jumps between 1917 and 1785 Ω (Fig. 1f), coinciding with the *R*–*I* curve in Fig. 1c. The resistance leaps without visible delay after the abrupt setting of current, and keeps constancy when the current is stable. Hence, the resistance change can be regarded as a state-dependent effect with time stability, *i.e*., the resistance is determined by the value of current rather than its variation.

The sample was also cooled to 100 K with a field of 1 T vertical to the electrical current (in-plane) and perpendicular to the film plane, respectively (case II and III in Fig. 1e). Corresponding *R*–*I* curves are presented in Fig. 1c. The magnitude of electroresistance in the three cases follows the order of case I ~ case III 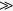 case II, presenting an anisotropic behavior. The field-cooling process excludes isotropic effects such as substrate morphology, tunneling at grain boundaries, and domain wall motions, and reflects the anisotropic AFM order parameters. In addition, the resistance reduction cannot be ascribed to neither current-induced Joule heating nor the magnetic proximity effect (MPE) in Pt induced by the interfacial uncompensated moments in FeMn^24^, because the rising temperature only leads to increasing resistance (Fig. 1a), while the MPE generally causes at most several-Ohm shift of the total resistance (Supplementary Figure S4).

To confirm the critical role of attached non-magnetic metal on the electroresistance, pure Al with weak spin-orbit coupling was chosen on purpose, and Al(4)/FeMn(7) sample was prepared. Figure 1a shows that the resistance of Al/FeMn at 100 K is nearly two times larger than Pt/FeMn, which suggests that Al might be partly oxidized. Different from Pt with strong spin-orbit coupling, the mix of Al and AlO_x_ does not generate any spin injection. The *R*–*T* curve in Fig. 1a has a turning point at 170 K, because the electrical conductance of the partly oxidized Al is smaller than Pt and cannot totally cover the negative *R*–*T* correlation of FeMn. Here we note that some dilute magnetic alloys such as FeMn and IrMn show anomalously negative temperature coefficient of resistivity due to the intense scattering of conducting electrons^28^,^29^. The *R*–*I* curves at 300 and 100 K are displayed in Fig. 1b,d, respectively. Besides the constant resistance at 300 K, the resistance in the three cases at 100 K has no difference but a weak reduction of 8 Ω, which is just a temperature effect caused by the current. This negligible resistance change indicates that magnetic microstructures in FeMn, the current-induced AFM domain structures in particular, cannot determine the resistance drop in Pt/FeMn by itself. In this scenario, spin injection from the non-magnetic metal to FeMn is expected to be responsible for the electroresistance.

**Figure 2:**
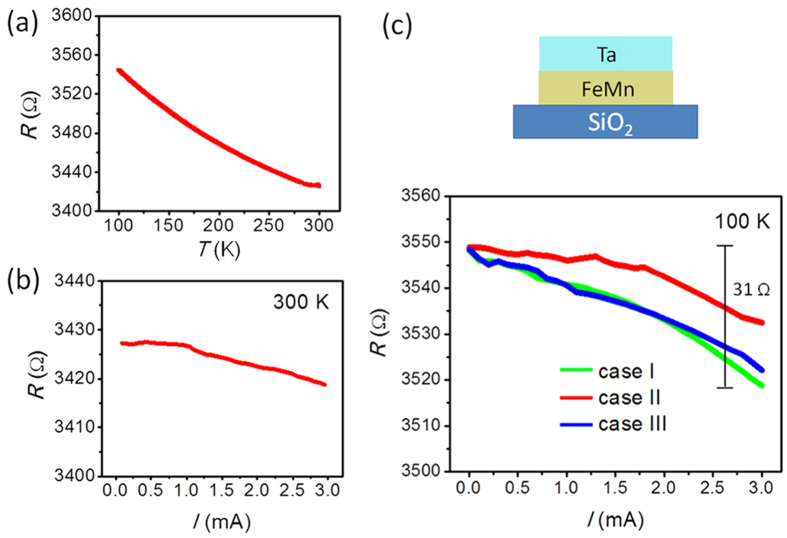
Electroresistance in Ta(4)/FeMn(7). (**a**) *R*–*T* curve measured with an applied current of 100 μA. (**b**) *R*–*I* curve measured at 300 K. (**c**) *R*–*I* curves after field cooling from 300 to 100 K with a magnetic field of 1 T. The data were measured at 100 K after repealing the cooling field. The field cooling modes are defined in Fig. 1(e).

Besides Pt, Ta is commonly used as an electrode for the transition between charge current and spin current. Particularly, the spin Hall angle of Ta has an opposite sign of Pt, meaning that the spins arriving at Ta/FeMn interface are opposite to those at Pt/FeMn interface^30^,^31^,^32^. The negative *R*–*T* relation of Ta(4)/FeMn(7) (Fig. 2a) derives from the negative temperature coefficient of both Ta and FeMn. The band structure change induced by lattice strain at low temperature and thermally activated electron transport process are proposed to contribute to the resistance decrease in Ta^33^. Different from the slight resistance drop when the current increases at 300 K (Fig. 2b), at 100 K the resistance shows negative relationship with the current in three cases (case I ~ case III > case II) (Fig. 2c), which is similar to the scenario of Pt/FeMn and performs no response to the negative spin Hall angle of Ta. The magnitude of electroresistance in case I (31 Ω) is nearly one fifth of that in Pt/FeMn, in agreement with the fact that the spin Hall angle of Ta is smaller than Pt^30^,^31^,^32^ (see Supplementary Information). This control experiment in Ta/FeMn suggests that the electroresistance is determined by the strength of spin current rather than the specific spin polarization injected to FeMn.

**Figure 3:**
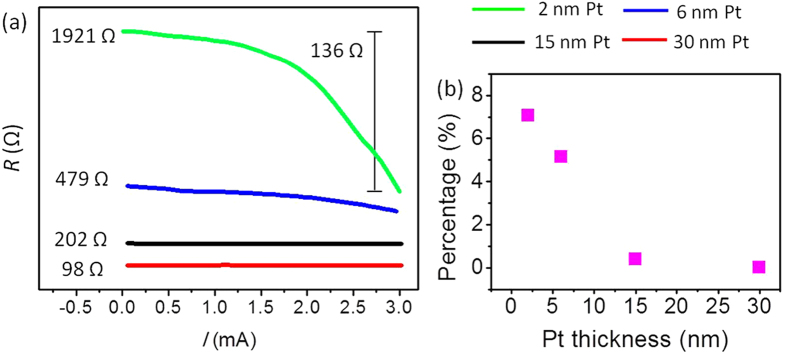
Thickness-depedent electroresistance of Pt/FeMn at 100 K in case I. (**a**) *R*–*I* curves of Pt(*t*)/FeMn(7) samples with different Pt thickness. (**b**) Summary of the relationship between the electroresistance percentage (defined as [*R*(*I* = 3 mA) − *R*(*I* = 0.005 mA)]/*R*(*I* = 0.005 mA), calculated from the results in (**a**)) and the Pt thickness.

To further demonstrate the SHE-induced spin injection, we investigate a series of Pt(*t*)/FeMn(7) samples with various thickness of Pt (*t* = 2, 6, 15, and 30 nm). The electroresistance percentage, defined as [*R*(*I* = 3 mA) − *R*(*I* = 0.005 mA)]/*R*(*I* = 0.005 mA), is measured at 100 K in case I. As plotted in Fig. 3 the contribution of the electroresistance to the total resistance becomes smaller with thicker Pt. This is because the spin relaxation length of Pt is around 1.5 nm^34^,^35^. In Pt with larger thickness, only the charge current near the Pt/FeMn interface in Pt can be converted to spin current via the SHE. In this way, the thickness-dependent results could provide a support to the SHE scenario.

**Figure 4:**
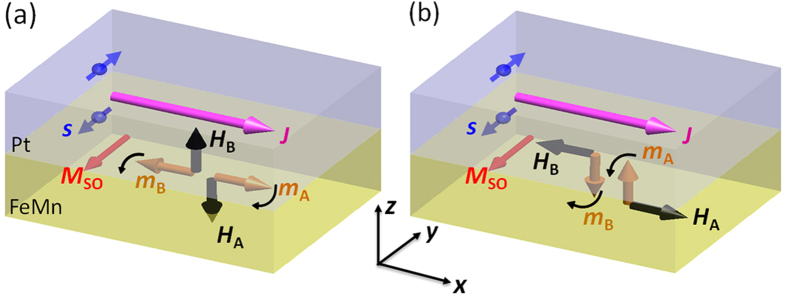
Antidamping-like SOT in Pt/FeMn. ***J*** is the applied charge current flowing through the bilayer. ***s*** represents the spin magnetic moment of the spins arriving at the Pt/FeMn interface and then injected to FeMn via the spin Hall effect in Pt. ***m***_A_ and ***m***_B_ represent the antiparallel magnetic moments in FeMn, The effective fields acting on ***m***_A_ and ***m***_B_ are symbolized by ***H***_A_ and ***H***_B_, respectively. The rotation trend of the AFM moments is shown by the curved arrows. ***M***_SO_ is the SOT-induced effective magnetization in FeMn. The AFM moments ***m***_A_ and ***m***_B_ are along (**a**) *x*-axis and (**b**) *z*-axis.

According to the experimental results above, the spin injection from the neighboring metal and the AFM order parameters in FeMn contribute together to the electroresistance in Pt/FeMn and Ta/FeMn. This rationally points to the category of spin-related torques. We ascribe the torque discussed in this work to the SOT, because the spin injection derives from the spin-orbit coupling in the neighboring metal^17^,^20^. In the case plotted in Fig. 4a, antiparallel AFM moments***m***_A_ and ***m***_B_ are dominantly along *x*-axis. Although the magnetic sublattices in FeMn are complicated and generally described by the 3Q model^36^, the field-cooling process can induce almost parallel and antiparallel FeMn moments to the cooling field^9^,^10^,^11^,^24^,^25^. A flow of spin current is injected from Pt to FeMn by applying an in-plane charge current (***J***) via the spin Hall effect. The spins injected to FeMn induce non-zero carrier spin-polarization which is parallel to the spin magnetic moment ***s*** and, as a result, an antidamping-like torque d***m***_A,B_/d*t* ~ ***m***_A,B_ × (***m***_A,B_ × ***s***)^14^,^37^,^38^. Forced by the SOT, the AFM moments turn to rotate towards –*y* direction, thus having relatively uncompensated alignment. An effective magnetization term ***M***_SO_ is introduced to describe this effect^39^. Similarly, if the AFM moments are aligned collinear with ***s*** along *y*-axis, no field-like SOT can be induced because ***m***_A,B_ × ***s*** = 0. And when the AFM moments are along *z*-axis (Fig. 4b), ***M***_SO_ can be found parallel to that in Fig. 4a.

By looking into the spin Hall magnetoresistance in heavy metal/FM hybrids^34^,^35^,^39^,^40^,^41^,^42^, the negative electroresistance in Pt/FeMn is found to have some similarity with the SMR. In case I and III, the torque exerted from the injected spin leads to ***M***_SO_//***s***, which results in the reflection of the Pt electron and the resultantly reduced resistance. Given that the magnitude of spin injection and ***M***_SO_, which are coupled in forming the SMR, are positively related to ***J***, negative electroresistance could be detected showing a non-linear relationship with the increasing current. The situation changes in case II where the injected spin is collinear the AFM moments. Neither the torque nor ***M***_SO_ is exerted, corresponding to no apparent resistance change. We note that the maximal SMR reported in recent publications is up to 1% in heavy metal/FM metal hybrids^40^, much weaker than the electroresistance (7% of the total resistance), which suggests some interesting mechanisms beyond the traditional framework of SMR. We wish more theoretical work can make contribution to clarifying the quantitative relationship between the SHE-induced SOT and the transport phenomena.

Let us look back to the electroresistance in Pt/FeMn. The small resistance drop in case II could be explained by the fact that the AFM moments in FeMn are not strictly aligned along *y*-axis. The remained *x*-components would contribute to the resistance reduction. The electroresistance in Ta/FeMn also coincides with the proposed model qualitatively. Given that d***m***_A,B_/d*t* ~ ***m***_A,B_ × (***m***_A,B_ × ***s***), ***M***_SO_ is always parallel to the spins arriving at the interface no matter what the direction of ***s*** is (Supplementary Figure S6). Consequently, the continuous spin injection accompanied with ***M***_SO_ still leads to the non-equilibrium spin accumulation in FeMn, resulting in the resistance reduction in case I and III. The smaller resistance change in case II (15 Ω) derives from the temperature effects mainly and the residual *x*-components of FeMn moments.

**Figure 5:**
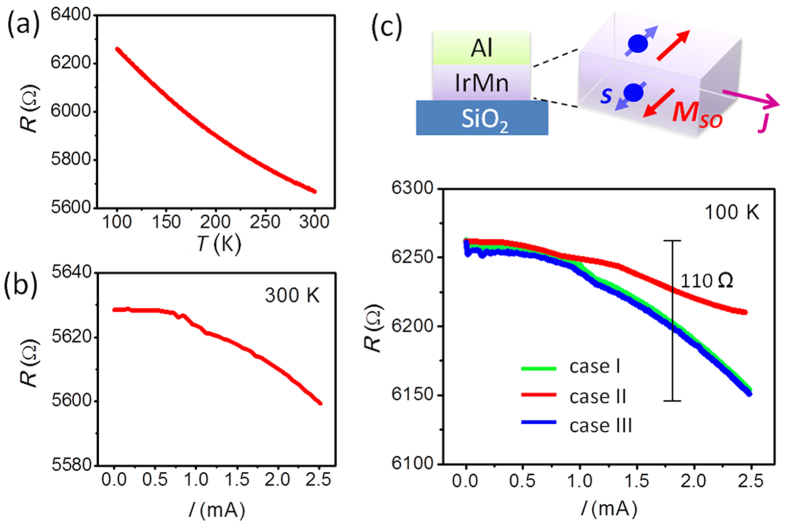
Electroresistance in Al(4)/IrMn(3). (**a**) *R*–*T* curve measured with an applied current of 100 μA. (**b**) *R*–*I* curve measured at 300 K. (**c**) *R*–*I* curve after field cooling from 300 to 100 K with a magnetic field of 1 T. The data were measured at 100 K after repealing the cooling field. The field cooling modes are defined in Fig. 1(e). A schematic of the spin Hall effect in IrMn is plotted in the upper right corner. ***J*** is the charge current through IrMn. ***s*** and ***M***_SO_ represent the local spin magnetic moment and effective magnetization at the interfaces of IrMn.

In the experiments above, the metals with spin-orbit coupling are indispensable for generating spin injection to FeMn. Given that the AFM alloy IrMn has bulk SHE to create carrier spin-polarization by itself^43^, the SOT in IrMn may be manipulated by dc current without assistance from the attached Pt. The *R*–*T* curve of Al(4)/IrMn(3) in Fig. 5a reveals the negative *R*–*T* characteristics of IrMn. A small resistance reduction of 25 Ω exists when the current increases at 300 K due to the Joule heating (Fig. 5b). The *R*–*T* curve with 1 T field is plotted in Supplementary Figure S7. After the sample is cooled to 100 K with a field of 1 T in case I and III, the resistance drops remarkably with the same magnitude (110 Ω) as the current rises up to 2.5 mA (Fig. 5c). In this scenario, the SHE in IrMn leads to spin accumulation at both the top and bottom interfaces of IrMn (see the schematic in Fig. 5c, which would result in effective magnetization parallel to the local spin polarization and thus contribute to the electroresistance. The smaller resistance reduction in case II (~40 Ω) can be mainly attributed to the temperature effects and the remained *x*-components of IrMn moments as well. Recently a new type of resistance, Hanle magnetoresistance, has been observed in heavy metals^44^. The spin accumulation created at the surfaces of the film by the SHE decreases in a magnetic field because of the Hanle effect, resulting in an increase of the electrical resistance. Since IrMn presents the SHE, it is expected to observe the Hanle magnetoresistance in plain IrMn.

## Discussion

We demonstrate the spin-Hall-effect-assisted electroresistance in antiferromagnetic FeMn and IrMn via 10^5^ A/cm^2^ dc current. The SHE-induced spin injection from neighboring Pt or Ta leads to the antidamping-like SOT in FeMn, while the SHE in IrMn generates the SOT by itself. The torque manipulates the spin transport process and leads to the remarkable resistance reduction. Besides fundamental insight into the SHE-induced SOT studied very recently, our work would open a promising avenue to purely AFM-based spintronic devices, and stimulate refined theoretical calculations for further understanding of the magneto-transport in metallic AFMs.

## Methods

### Sample preparation

The Pt/FeMn, Al/FeMn, Ta/FeMn, and Al/IrMn thin films were deposited on Si/SiO_2_ substrates by magnetron sputtering with highly pure Ar of 0.40 Pa at room temperature. The films were then fabricated into Hall-bars by standard lithography and Ar ion milling for four-probe measurements. The width of the Hall-bars is 50 μm, and the distance between the two side electrodes which were used to measure the longitudinal resistance (*R*) is 400 μm. The surface morphology was examined by the atomic force microscopy.

### Transport measurements

We used four-probe measurements on patterned Pt/FeMn, Ta/FeMn, and Al/IrMn Hall-bars. An electrical current of 100 μA was applied along the longitudinal direction to measure the resistance–temperature curves of these samples. The current was increased to detect the current dependence of resistance. The applied current *I* was supplied by B2901A Precision Source/Measure Unit, and the longitudinal resistance *R* was measured by 34100A 6^1^/_2_ Digit Multimeter.

## Additional Information

**How to cite this article**: Han, J. *et al*. Spin-Hall-Effect-Assisted Electroresistance in Antiferromagnets via 10^^5^^  A/cm^2^ dc Current. *Sci. Rep*. **6**, 31966; doi: 10.1038/srep31966 (2016).

## Supplementary Material

Supplementary Information
