# Supplementary material for: Spin-Hall-Effect-Assisted Electroresistance in Antiferromagnets via 105 A/cm2 dc Current
Source: Sci Rep. 2016 Aug 22;6:31966. doi: 10.1038/srep31966 (PMC4992953; doi:10.1038/srep31966)
Supplement: Supplementary Information [file srep31966-s1.doc]

**Supplementary Information for**

**Spin-Hall-Effect-Assisted Electroresistance in Antiferromagnets via 10^5^ A/cm^2^ dc Current**

Jiahao Han, Yuyan Wang, Feng Pan, and Cheng Song

Correspondence to: songcheng@mail.tsinghua.edu.cn (C.S.)

1. **Antiferromagnetism and surface morphology of the samples**

We first investigate the antiferromagnetic order of FeMn. Multilayers containing Pt(2)/FeMn(7)/Pt(0.6)Co(0.5)[Pt(1)/Co(0.5)]_4_Pt(8) (from top to bottom) were grown on Si/SiO_2_ substrates (unit of thickness: nanometer) and then made to be Hall-bars. A cooling field of 1 T was applied perpendicularly to the film plane at 100 K before the measurements. The transverse Hall resistance (*R*_H_) was measured under out-of-plane magnetic fields to reveal the magnetization of the sample via the anomalous Hall effect^S1^. The exchange bias appears is ~5 mT at 100 K and 0 at 200 K (Figure S1a and b), indicating the blocking temperature of 7 nm FeMn is between 100 and 200 K. The results also confirm the perpendicular exchange interactions of 7 nm FeMn at 100 K.

The effective alignments of the FeMn moments can be reflected by the different resistance created by the cooling fields. According to the data in Figure 1c, with a small current of 0.01 mA, the resistance of Pt(2)/FeMn(7) sample at 100 K appears to be 1919.46, 1920.70, and 1920.76 Ω for case I, II, and III, respectively. This anisotropic behavior of resistance (case I < case II ~ case III) suggests that the AFM moments have similar orientations with the electrical current in case II and III (perpendicular), which is different from case I (parallel). This indicates that the field cooling from 300 to 100 K can effectively align the FeMn moments.

To estimate the FM phase contribution in FeMn, we measured the anomalous Hall resistance of Pt(2)/FeMn(7) sample with a perpendicular sweeping field at 100 K. The results are plotted in Figure S1c in the Supporting Information. The Hall resistance is almost linear with the external field and shows no hysteresis, which is just an original Hall effect caused by the Lorentz force. Compared with the significant anomalous Hall effect in [Co/Pt]/FeMn sample (Figure S1a), the FM phase in Pt(2)/FeMn(7) can be neglected. In addition, plain FeMn has no response to external field in X-ray magnetic linear dichroism (XMLD) signals, in contrast to the case of [Co/Pt]/FeMn hybrids, which suggests the negligible FM components in FeMn^S2^.

We also use the temperature dependence of the electroresistance to reflect the AFM order in FeMn. The magnitude of the electroresistance *ER*=*R*(*I*=3mA)–*R*(*I*=0.005mA) of Pt(2)/FeMn(7) is measured from 100 to 300 K in case I. As plotted in Figure S1d, the electroresistance has moderate change from 100 to 180 K and decreases significantly between 180 and 220 K, which coincides with the AFM transition of FeMn. The temperature of the sharp decrease in turn demonstrates the interactions between the injected spins and the AFM moments in FeMn for generating the electroresistance.

The surface morphology of the Pt(2)/FeMn(7) sample is investigated by the atomic force microscopy (Figure S2b). The roughness of the surface is 0.58 nm, which is endurable for transport studies in the sample with the thickness of 9 nm in total.


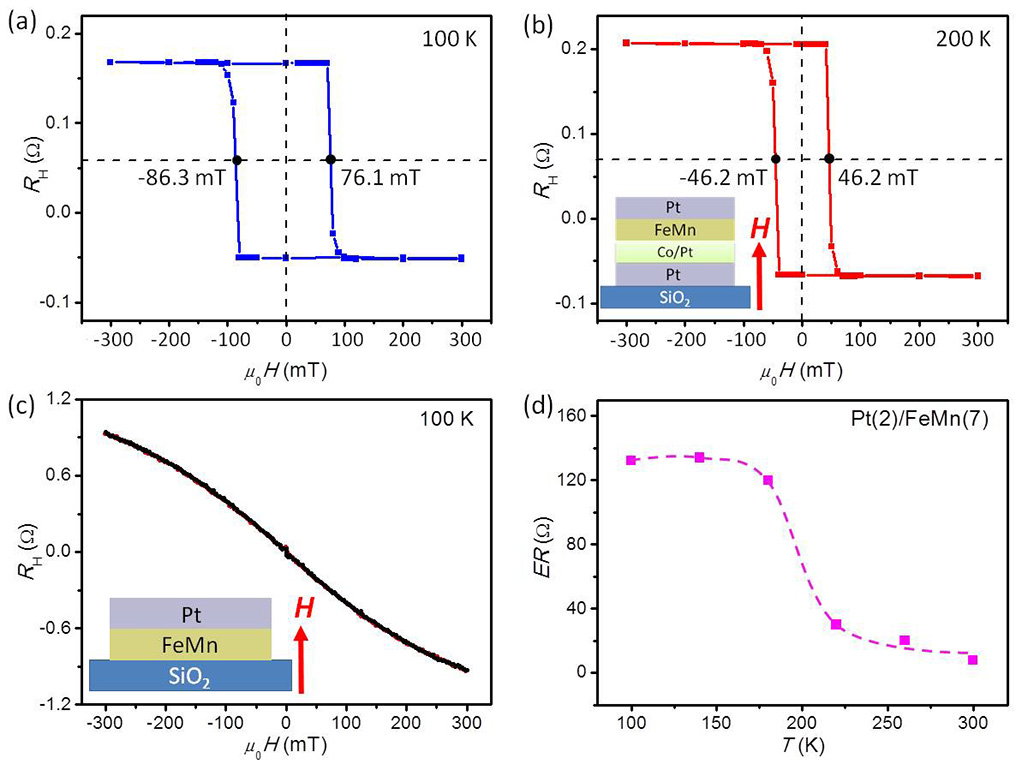


Figure S1. (a) and (b) Exchange bias reflected by the anomalous Hall resistance in Pt(2)/FeMn(7)/Pt(0.6)Co(0.5)[Pt(1)/Co(0.5)]_4_Pt(8) (from top to bottom) measured at 100 and 200 K, respectively. The sweeping magnetic field is perpendicular to the film plane. (c) Anomalous Hall resistance in Pt(2)/FeMn(7) measured at 100 K. (d) Temperature dependence of the electroresistance in Pt(2)/FeMn(7) in case I.


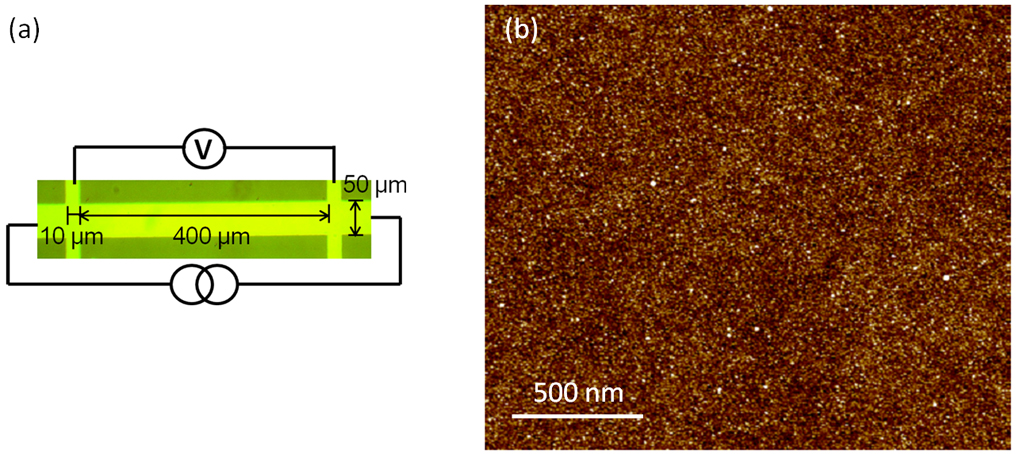


Figure S2. (a) Optical image of the Hall-bar used in our experiments with an electrical current applied along the longitudinal direction to measure the longitudinal resistance. (b) Image of the surface morphology of Pt(2)/FeMn(7) on Si/SiO_2_ substrate measured by the atomic force microscopy.

1. **Effects of Ørsted fields and Joule heating generated by the dc current**

Because of the channel width (*w*) of 50 μm and the film thickness of several nanometers, the films patterned as Hall-bars can be regarded as an infinite plane^S3^. The relation between magnetic field *μ*_0_*H* and current *J* is *μ*_0_*H* = *μ*_0_*J*/2*w*. The maximum Ørsted field is thus ~0.04 mT generated by the electrical current of 3 mA, which is too subtle to take into consideration.

We next examine the temperature effects of the applied current. Previous work suggests that an electrical current of 3 mA flowing through a Hall-bar with the width of 50 μm induces a temperature increase of ~9 K due to the Joule heating^S4^. From this we can further clarify the influence of Joule heating on the resistance changes.

(1) Al/FeMn. As displayed in Figure 1a, the resistance of Al/FeMn decreases by ~6 Ω when the temperature rises from 100 to 110 K. In this case, the temperature increase of 10 K can be equivalent to an electrical current of 3 mA. Hence, the resistance reduction of 7~8 Ω in Figure 1d should be attributed to the current-induced Joule heating.

(2) Ta/FeMn. A detailed study of Figure 2a shows that the temperature increase from 100 to 110 K leads to a resistance reduction of 9 Ω, which is quite smaller than that in Figure 2c (31Ω). Thus, the resistance changes in Figure 2c, especially in case I and III, should not be ascribed to Joule heating only.

(3) Al/IrMn. Figure 5a shows that the resistance decreases by 25 Ω when the temperature increases by 10 K near 300 K, corresponding to the current-induced resistance reduction at 300 K in Figure 6b.

1. **Details of the current-induced electroresistance in Pt/FeMn**

To confirm the current-induced electroresistance in Pt/FeMn, we also carried out same experiments using another Pt(2)/FeMn(7) sample fabricated in the same condition as the sample discussed in the main text. The *R*–*I* curves at 100 K are plotted in Figure S3a. The data have similar trend with the results in the main text, which suggest the reproducibility of the current-induced electroresistance in Pt/FeMn. The coincidence of the blue line in Figure S3a can be interpreted as follows. Most AFMs including FeMn and IrMn have in-plane easy axis. Moreover, influenced by the shape of the Hall-bars (Figure S2a, length >> width >> thickness), the AFM moments partly have some priority to arrange in the film plane. Hence, the AFM moments have a dominant arrangement along x-axis in case I. Comparatively, in case III, although the main arrangement of the AFM moments is forced to be along z-axis, residual in-plane components may still exist.

The resistance of Pt(2)/FeMn(7) in case I at 100 K with negative current is plotted in Figure S3b, which presents very similar behaviors as the positive current. This is because the compensated alignment of AFM moments does not break the symmetry along *x*-axis.

We also measured the electroresistance when the cooling field of 1 T was maintained. There is almost no difference between the cases with and without the external field. Here we note that the interactions between magnetic moments and the internal spins should not be regarded equally to those between moments and external fields. The electroresistance derives from the non-equilibrium spin accumulation at Pt/FeMn interface. It is sensitive to the SHE-induced spin injection but rigid to external fields which do not generate any additional spin injection. This effect is similar to the electrical switching of the AFM CuMnAs, where the switching of AFM moments derives from the current-induced SOT but robust against magnetic field perturbations^S5^. As discussed above, the internal interactions between spins and magnetic moments could be much more significant than the effects of external fields.


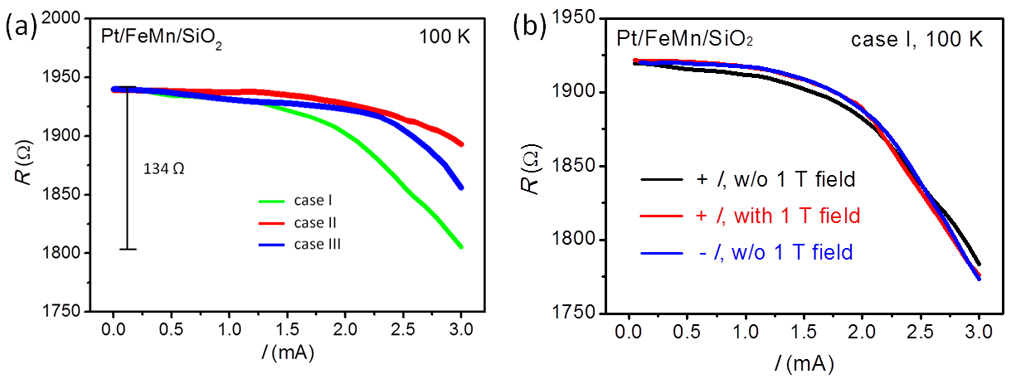


Figure S3. (a) *R*–*I* curves of another Pt(2)/FeMn(7) sample (fabricated in the same condition as the sample discussed in the main text) after field cooling from 300 to 100 K with a magnetic field of 1 T. The data were measured at 100 K after repealing the cooling field. (b) *R*–*I* curves with positive current and zero field (black curve), positive current and 1 T field (red curve), and negative current and zero field (blue curve) of Pt(2)/FeMn(7) measured in case I at 100 K.

1. **Influence of magnetic proximity effect in Pt**

Due to the possible existence of uncompensated AFM moments at Pt/FeMn interface, the magnetic proximity effect (MPE) might occur in Pt and affect the magneto-transport^S6^. We replaced FeMn by a ferromagnetic alloy NiFe, where the MPE does appear at the Pt/NiFe interface^S7^. We measured the current-dependent resistance of Pt(2)/NiFe(7) at 300 K (lower than the Curie temperature of NiFe) after NiFe was magnetized along the current. The resistance change is less than 3 Ω no matter NiFe is saturate magnetized or not magnetized at all (Figure S4). The small resistance change in NiFe should be related to current-induced Joule heating, which suggests that the MPE at the Pt/magnet cannot lead to the electroresistance in Figure 1c. Moreover, the remarkable electroresistance was also observed in plain IrMn films without the attached Pt, thus excluding the influence of the MPE.

**
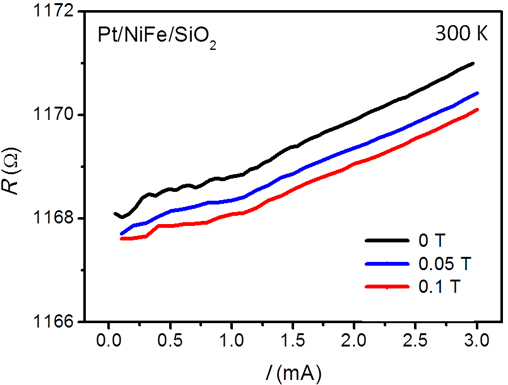
**

Figure S4. Current-induced resistance change in Pt(2)/NiFe(7) after NiFe is magnetized by the magnetic fields of 0, 0.05, and 0.1 T along the direction of applied current**.** The magnetization and the measurements were carried out at 300 K.

1. **Spin Hall angle of Ta**

The spin Hall angle of Ta depends on its phase. Beta phase Ta with high resistivity shows large spin Hall angle^S8^. We measured the SMR in Ta(4 nm)/YIG at 100 K, shown in Figure S5. The resistivity of Ta is calculated to be 26 μΩcm, much smaller than the typical resistivity of beta phase Ta. On the other hand, the SMR ratio in Ta(4 nm)/YIG is 1.3×10^-4^, even smaller than that of Pt(3,5 nm)/YIG at 300 K (3.8×10^-4^ and 2.4×10^-4^ for 3 and 5 nm samples)^S3^. Given that the spin Hall angle is the dominant parameter of the SMR ratio, it indicates that the spin Hall angle of Ta is smaller than Pt. Hence, we can confirm that the Ta used in our work has weaker spin Hall angle than Pt.


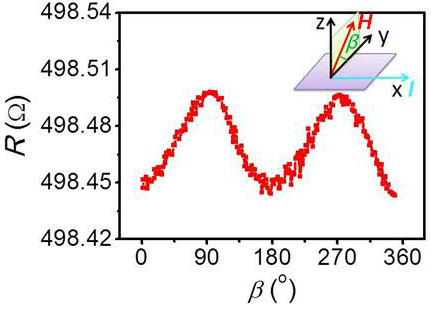


Figure S5. Spin Hall magnetoresistance in Ta(4 nm)/YIG at 100 K. The angle *β* is defined in the schematic at the corner.

1. **Antidamping-like spin-orbit torque in Ta/FeMn**

**
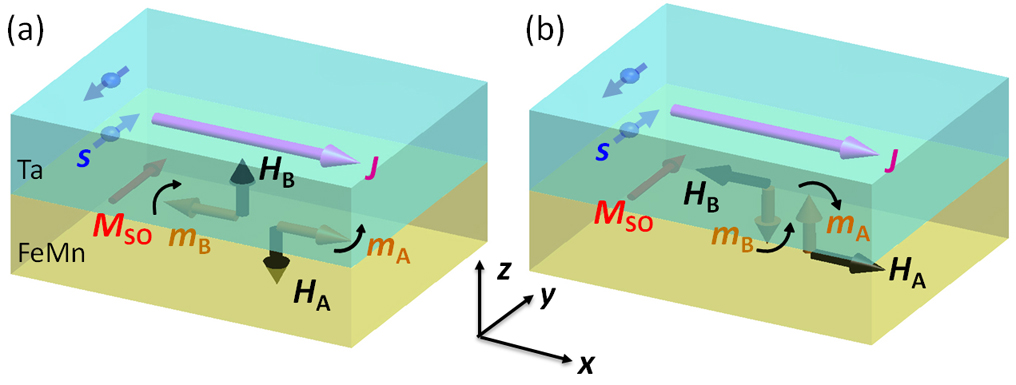
**

Figure S6. Antidamping-like SOT in FeMn attached by Ta. ***J*** is the applied charge current flowing through the bilayer. ***s*** represents the spin magnetic moment of the spins arriving at the Ta/FeMn interface and then injected to FeMn *via* the spin Hall effect in Ta. ***m***_A_ and ***m***_B_ represent the anti-parallel magnetic moments in FeMn, the effective fields on which are symbolized by ***H***_A_ and ***H***_B_, respectively. The rotation trend of the AFM moments is shown by the curved arrows. ***M***_SO_ is the SOT-induced effective magnetization in FeMn. The AFM moments ***m***_A_ and ***m***_B_ are along (a) *x*-axis and (b) *z*-axis.

1. **Resistance in Al/IrMn with 1 T field at 300 K**


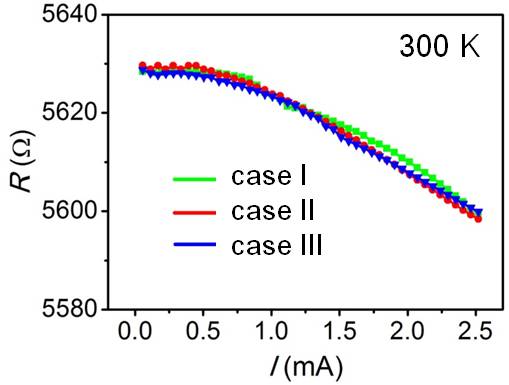


Figure S7. *R*–*I* curve of Al/IrMn with 1 T field in three cases measured at 300 K.

**References**

1. Wang, Y. Y. *et al*. [Electrical control of the exchange spring in antiferromagnetic metals](http://onlinelibrary.wiley.com/doi/10.1002/adma.201405811/full). *Adv. Mater.* **13**, 3196–3201 (2015).
2. Wang, Y. Y., Song, C., Wang, G. Y., Zeng, F. & Pan, F. Evidence for asymmetric rotation of spins in antiferromagnetic exchange-spring. *New J. Phys.* **16**, 123032 (2014).
3. Han, J. H. *et al*. Realization of the meminductor. *ACS Nano* **8**, 10043–10047 (2014).
4. Lin, D. C. *et al*. [Giant coercivity in perpendicularly magnetized cobalt monolayer](http://scitation.aip.org/content/aip/journal/apl/101/11/10.1063/1.4752446). *Appl. Phys. Lett.* **101**, 112405 (2012).
5. Wadley, P. *et al*. Electrical switching of an antiferromagnet. *Science* **351**, 587–590 (2016).
6. Kosub, T., Kopte, M., Radu, F., Schmidt, O. G. & Makarov, D. [All-electric access to the magnetic-field-invariant magnetization of antiferromagnets](http://journals.aps.org/prl/abstract/10.1103/PhysRevLett.115.097201). *Phys. Rev. Lett.* **115**, 097201 (2015).
7. Lu Y. M. *et al*. Hybrid magnetoresistance in the proximity of a ferromagnet. *Phys. Rev. B* **87**, 220409(R) (2013).
8. Liu, L. *et al*. Spin-torque switching with the giant spin Hall effect of tantalum. *Science* **336**, 555–558 (2012).
